# Deltex1 is inhibited by the Notch–Hairy/E(Spl) signaling pathway and induces neuronal and glial differentiation

**DOI:** 10.1186/s13064-015-0055-5

**Published:** 2015-12-30

**Authors:** Yi-Chuan Cheng, Yin-Cheng Huang, Tu-Hsueh Yeh, Hung-Yu Shih, Ching-Yu Lin, Sheng-Jia Lin, Ching-Chi Chiu, Ching-Wen Huang, Yun-Jin Jiang

**Affiliations:** Graduate Institute of Biomedical Sciences, College of Medicine, Chang Gung University, 259 Wen-Hwa 1 Road, Taoyuan, 33383 Taiwan; Neuroscience Research Center, Chang Gung Memorial Hospital at Linkou Medical Center, Taoyuan, Taiwan; Department of Neurosurgery, Chang Gung Memorial Hospital, Chiayi branch, Chiayi, Taiwan; College of Medicine, Chang Gung University, Taoyuan, Taiwan; Section of Movement Disorders, Department of Neurology, Chang Gung Memorial Hospital at Linkou Medical Center, Taoyuan, Taiwan; Institute of Molecular and Genomic Medicine, National Health Research Institutes, Zhunan Town, Miaoli County Taiwan

**Keywords:** Dtx1, Neural differentiation, Notch, Hairy/E(Spl), Zebrafish

## Abstract

**Background:**

Notch signaling has been conserved throughout evolution and plays a fundamental role in various neural developmental processes and the pathogenesis of several human cancers and genetic disorders. However, how Notch signaling regulates various cellular processes remains unclear. Although Deltex proteins have been identified as cytoplasmic downstream elements of the Notch signaling pathway, few studies have been reported on their physiological role.

**Results:**

We isolated zebrafish *deltex1* (*dtx1*) and showed that this gene is primarily transcribed in the developing nervous system, and its spatiotemporal expression pattern suggests a role in neural differentiation. The transcription of *dtx1* was suppressed by the direct binding of the Notch downstream transcription factors Her2 and Her8a. Overexpressing the complete coding sequence of Dtx1 was necessary for inducing neuronal and glial differentiation. By contrast, disrupting Dtx1 expression by using a Dtx1 construct without the RING finger domain reduced neuronal and glial differentiation. This effect was phenocopied by the knockdown of endogenous Dtx1 expression by using morpholinos, demonstrating the essential function of the RING finger domain and confirming the knockdown specificity. Cell proliferation and apoptosis were unaltered in Dtx1-overexpressed and -deficient zebrafish embryos. Examination of the expression of *her2* and *her8a* in embryos with altered Dtx1 expression showed that Dxt1-induced neuronal differentiation did not require a regulatory effect on the Notch–Hairy/E(Spl) pathway. However, both Dtx1 and Notch activation induced glial differentiation, and Dtx1 and Notch activation negatively inhibited each other in a reciprocal manner, which achieves a proper balance for the expression of Dtx1 and Notch to facilitate glial differentiation. We further confirmed that the Dtx1–Notch–Hairy/E(Spl) cascade was sufficient to induce neuronal and glial differentiation by concomitant injection of an active form of Notch with *dtx1*, which rescued the neuronogenic and gliogenic defects caused by the activation of Notch signaling.

**Conclusions:**

Our results demonstrated that Dtx1 is regulated by Notch–Hairy/E(Spl) signaling and is a major factor specifically regulating neural differentiation. Thus, our results provide new insights into the mediation of neural development by the Notch signaling pathway.

**Electronic supplementary material:**

The online version of this article (doi:10.1186/s13064-015-0055-5) contains supplementary material, which is available to authorized users.

## Background

In the developing central nervous system, neural progenitor cells in the ventricular zone of the neural tube proliferate extensively and, following asymmetric cell division, generate neuronal and glial precursors that produce various types of neurons and glial cells. Generating these neural cells requires numerous gene regulatory and signaling processes, and understanding the regulatory mechanisms during development may provide crucial implications for developing repair therapies for treating nervous system injuries and tumors. Notch signaling has been conserved throughout evolution and plays a fundamental role in various neural developmental processes and the pathogenesis of several human cancers and genetic disorders [1]. In the developing nervous system, Notch signals are involved in neuronal progenitor maintenance, and they later control the differentiation of neuronal and glial lineages [2]. The transmembrane Notch receptor is activated on binding to the membrane-bound Delta or Serrate ligand present on an adjacent cell. This interaction triggers cleavage to release a cytoplasmic fragment of Notch that enters the nucleus and interacts with the DNA-binding protein CSL (CBF/RBP-J, Su(H), LAG-1/CSL), leading to the transcription of target genes such as Hairy and Enhancer-of-split [*Hairy/E(spl*)] [3].

Deltex has been identified as a cytoplasmic downstream element of the Notch signaling pathway [4, 5]. Deltex family members contain three conserved domains separated by blocks of glutamine-rich sequences. The N-terminal domain contains two WWE domains and is responsible for binding to the Notch intracellular domain. The middle section contains a proline-rich sequence that was proposed to be an SH3 domain-binding site, and the C terminus contains a RING zinc-finger motif [4]. Deltex regulates Notch signaling by physically interacting with the Notch intracellular domain independently of the canonical downstream CSL-Hairy/E(Spl) cascade [5]. Activating Deltex-dependent Notch signaling represses neural fate in Drosophila, suggesting that Deltex acts as a positive regulator of Notch signaling [6]. However, depending on developmental and cellular context, Deltex may also act as a negative regulator [7, 8]. The exact mechanism of Deltex-dependent Notch signaling remains unknown.

To date, four mammalian homologs, DELTEX1–4 (DTX1–4), have been identified [9, 10], and all four homologs share a high degree of sequence and structural similarity; however, few studies have been reported on their physiological role. A study of the neuroepithelial cell line MNS-70 showed that mouse DTX1 mediates Notch signaling, blocking neural progenitor cell differentiation [11]. DTX1 is also essential for the differentiation of oligodendrocytes in rat primary cell cultures [12]. The overexpression of mouse *Dtx1*, *Dtx2*, or *Dtx3* in *Xenopus* results in an expansion of the neuroepithelium [9]. In addition to the nervous system, DTX1 also regulates lineage commitment in lymphocytes [7, 13], and *Dtx1* knockout mice have shown T cell anergy [14]. In addition to their direct interaction with Notch, several in vitro studies have shown that Deltex proteins also interact with Wnt [6], Ras [15], and BMP [16] signaling. However, how Deltex-dependent transduction is achieved is poorly understood.

We isolated the first zebrafish *deltex* gene, which showed the highest sequence similarity to mammalian *DTX1*/*Dtx1*. Zebrafish *dtx1* was expressed in the developing nervous system, and *dtx1* transcription was inhibited by the direct binding of the Notch downstream transcription factors Her2 and Her8a on the *dtx1* promoter region. Overexpressing the complete coding sequence of Dtx1 induced neuronal and glial differentiation. By contrast, the overexpression of Dtx1 constructs lacking the RING finger domain inhibited neuronal and glial differentiation, and this dominant negative effect was confirmed by the knockdown of Dtx1 expression by morpholinos. Furthermore, we showed that Dxt1-induced neuronal and glial differentiation did not require a feedback regulation via the Notch–Hairy/E(Spl) pathway. Dtx1 did not regulate the Notch-HES/Her pathway during neuronal differentiation; on the contrary, Dtx1 reciprocally inhibited Notch during glial differentiation. Our data provide further insights into the roles of Dtx1 in neural development and Notch-regulated neural differentiation.

## Results

### Characterization of Zebrafish *dtx1*

Zebrafish *dtx1* comprised 1884 bp, encoding a 628-residue peptide containing putative start and stop codons matching approximately the same positions as in other vertebrate *DTX1*/*Dtx1* genes. Phylogenetic analysis showed that this fragment was most closely related to mammalian DTX1 (Additional file 1b). We therefore named this gene zebrafish *dtx1* (GenBank accession number KR869089). The zebrafish Dtx1 amino acid sequence showed that the structural features of the three domains, WWE, proline-rich, and RING finger, were conserved with other Deltex family members (Additional file 1a). Compared with other orthologs of the Deltex proteins, zebrafish Dtx1 showed the highest degree of similarity to mouse and human DTX1, with 74 % identical amino acids.

### Expression of *dtx1* in the developing nervous system

**Fig. 1:**
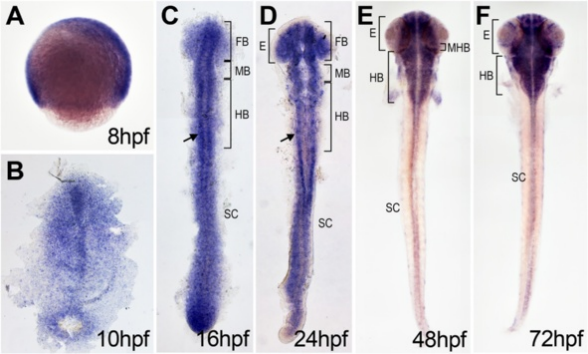
*dtx1* expression in developing zebrafish. *dtx1* expression was detected using *in situ* hybridization in the developing nervous system during zebrafish embryogenesis. The embryo stages are shown in the bottom right corner of each panel. **a** Lateral view with anterior to the right. **b**–**e** Dorsal view with anterior to the top. **b**–**f** *dtx1* expression occurred first in the developing nervous system during the bud stage (**b**) and remained until the final stage that was analyzed (**e**) Arrows in **c** and **d** indicate *dtx1* expressing cells flanking the midline. E, eye; FB, forebrain; HB, hindbrain; MB, midbrain; MHB, midbrain-hindbrain boundary; SC, spinal cord

The expression of *dtx1* was analyzed using whole-mount *in situ* hybridization. Transcripts first appeared in the developing nervous system at the bud stage in the primordium of the brain and spinal cord (Fig. 1b). From the midsegmentation stages, cells with different *dtx1* expression levels spanned the entire central nervous system, with strong expression in cells flanking the midline (arrows in Fig. 1c,d). This expression remained until the late pharyngula stages (48 hpf; Fig. 1e). Abundant *dtx1* expression was also detected in the entire brain but not in the mid-hindbrain boundary at 48 hpf (Fig. 1e). In general, the expression pattern of zebrafish *dtx1* was similar to that of mouse *Dtx1*, which is also expressed in the developing central nervous system [9, 17].

### Role of the Delta–Notch–Hairy/E (Spl) Signaling Pathway in *dtx1* Regulation

**Fig. 2:**
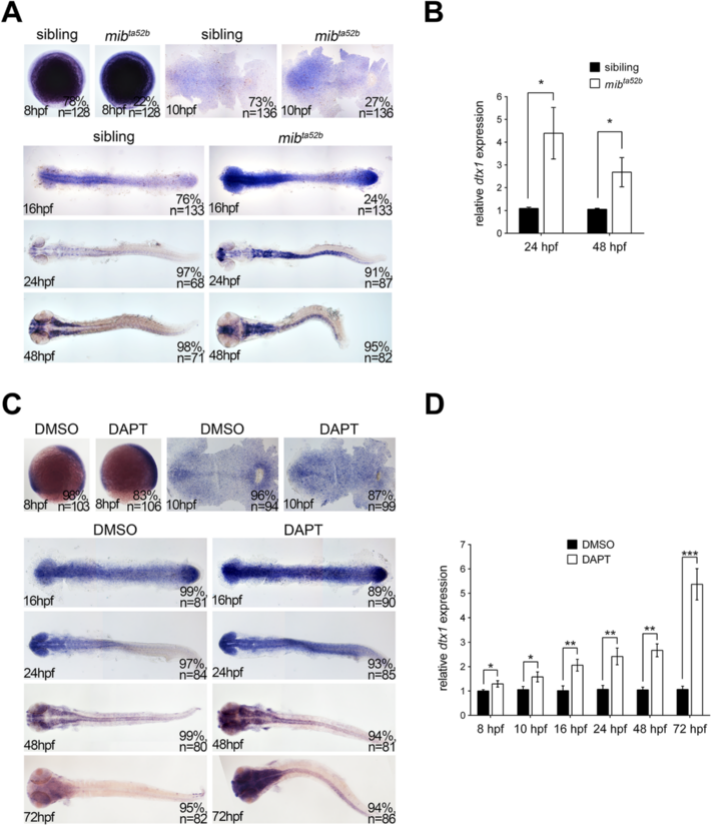
*dtx1* expression is upregulated in Notch-deficient embryos. **a** The expression of *dtx1* was analyzed in *mib* ^*ta52b*^ homozygous mutants and wild-type siblings through *in situ* hybridization. The stages are shown in the bottom left corner. Embryos were produced by crossing the parents with heterozygous mutant genotype. Embryos at 8 hpf, 10 hpf, and 16 hpf stages were mixed genotypes containing heterozygous *mib* ^*ta52b*^ mutation, homozygous *mib* ^*ta52b*^ mutation, and wild-type *mib*, and they were grouped according to the substantial difference in *dtx1* expression. The results indicate that approximately 75 % of wild-type and heterozygous *mib* ^*ta52b*^ siblings had unaltered *dtx1* expression, whereas approximately 25 % of homozygous *mib* ^*ta52b*^ mutant*s* had increased *dtx1* expression. Embryos at 24 hpf and 48 hpf were grouped according the morphological defects that only appeared in *mib* ^*ta52b*^ homozygous mutants. **c**. DAPT treatment was performed at different time points, and the embryos were harvested at different stages as indicated; the results show that DAPT treatment caused upregulation of *dtx1* expression. **b** and **d** The results of *in situ* hybridization in **a** and **c** were quantitatively confirmed using qPCR analysis, respectively. *, *P* < 0.05

A previous study showed that the constitutively active form of Notch upregulated *Dtx1* expression in a mouse thymoma cell line [18]. To examine the response of *dtx1* to Notch signaling in the developing nervous system, we analyzed its expression in *mind bomb* mutant embryos (*mib*^*ta52b*^), which have a deficient Notch pathway [19] because of a missense mutation in the most C-terminal RING domain that bears ubiquitin ligase activity required for Delta ligand internalization and Notch activation [20]. Compared with wild-type embryos, *dtx1* expression in the developing nervous system was considerably upregulated in *mib*^*ta52b*^ embryos (Fig. 2), suggesting that Delta–Notch signaling activation is essential for inhibiting *dtx1* transcription. This result was further confirmed by the γ-secretase inhibitor, N-[N-(3,5-difluorophenacetyl)-L-alanyl]-S-phenylglycine t-butyl ester (DAPT), which has been shown to efficiently block Notch signaling at different time-points [21]. Embryos were treated with DAPT at the shield stage (6 hpf) and harvested at 75 % epiboly (8 hpf), at 8 hpf and harvested at the bud stage (10 hpf), 24 hpf, or 48 hpf. The embryos that were treated with DAPT at different time-points exhibited an upregulation of *dtx1* expression to a level similar to that observed in the *mib*^*ta52b*^ mutant (Fig. 2b,d), which demonstrated that Notch signaling was essential for the inhibition of *dtx1* expression. The converse regulatory mechanism between the thymoma cell line and developing nervous system suggested that the regulation of *Dtx1*/*dtx1* expression by Notch signaling is highly dependent on the cell type.

**Fig. 3:**
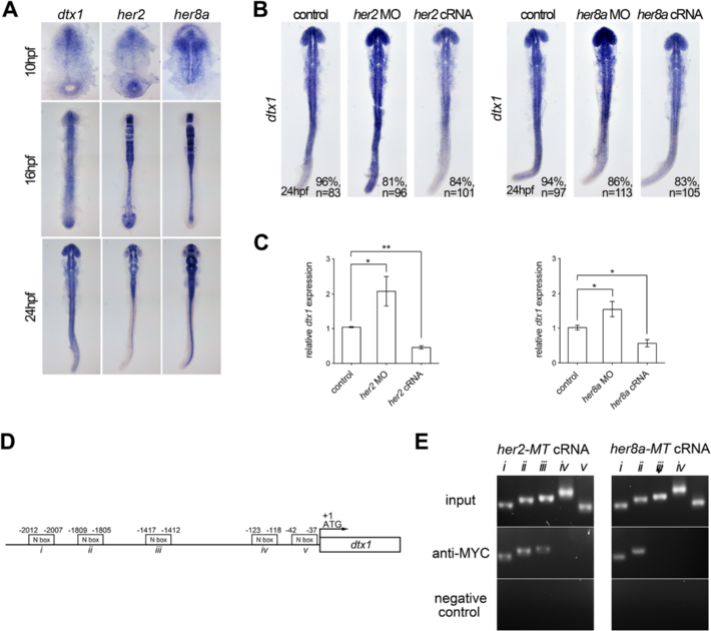
Her2 and Her8a inhibits *dtx1* transcription through direct binding to the promoter region. **a** Comparison of the expression of *dtx1* with *her2* and *her8a* showing partially overlapped expression in the developing nervous system. **b** *In situ* hybridization result showing that *her2* or *her8a* cRNA inhibited *dtx1* expression. **c** qPCR results showing that *her2* or *her8a* cRNA injection downregulated *dtx1*expression. *, *P* < 0.05; **, *P* < 0.01. **d** A schematic representation of the promoter region of *dtx1*, and fragments containing potential N-boxes are named and indicated. These fragments were selected for PCR amplification. **e** Chromatin immunoprecipitation (ChiP)–PCR analysis was performed on the bud stage embryos. Many fragments (i, ii, and iii) that were amplified by PCR suggested the direct binding of Her2 and Her8a to these regions

The transcription factors Hairy/E(Spl) (named HES in mammals and Her in zebrafish) are major downstream targets of Notch signaling, and they suppress the transcription of proneural genes, resulting in the inhibition of neuronal differentiation [3]. In particular, HES1 and HES5 are essential for the inhibition of neurogenesis and to promote the differentiation of many glial subtypes [1, 2]. Therefore, we examined whether *dtx1* transcription is directly regulated by Hairy/E(Spl) transcription factors. We have demonstrated that the zebrafish Hairy/E(Spl) homolog Her2 is a homolog of mammalian HES5, and that Her8a belongs to the HES6 group but functionally resembles mammalian HES5. Moreover, Her2 and Her8a are regulated by Delta–Notch signaling, and they mediate neurogenesis and gliogenesis [22, 23]. The expression of *her2* or *her8a* was partially overlapped with that of *dtx1* in the developing nervous system during neuronal differentiation, suggesting an interaction of Her2 and Her8a with Dtx1 (Fig. 3a). Accordingly, we examined whether Her2 and Her8a regulates *dtx1* expression. The results of qPCR analyses and *in situ* hybridization demonstrated that *her2* or *her8a* cRNA injections downregulated *dtx1* expression (Fig. 3b,c). Next, we performed a ChIP analysis to confirm the direct binding of Her2 and Her8a to *dtx1* promoter. We tagged Her2 and Her8a with a Myc tag and used an antibody against Myc in the ChIP assays on extracts from the bud stage embryos. After injecting *her2-myc* or *her8a-myc* cRNA into fertilized eggs, we detected high Myc levels (data not shown), and the chromatin fragments isolated from these embryos were immunoprecipitated with an antibody against the Myc tag. Studies have shown that mouse HES1 and zebrafish Her2 directly bind to N-boxes in the promoter regions of target genes [22, 24, 25]. Through direct sequence comparison, we identified several N-boxes in the *dtx1* promoter and accordingly designed PCR primers (Fig. 3d) to investigate whether the fragments from the ChIP were selectively amplified. The results revealed that both Her2 and Her8a bound to numerous N-boxes in the *dtx1* promoter (Fig. 3e). The results demonstrated that Her2 and Her8a repressed *dtx1* transcription by directly binding to the N-boxes of the promoter regions.

To further confirm the direct binding of Her2 and Her8a to the *dtx1* promoter, we created a fragment containing the Her2 and Her8a binding N-boxes of the *dtx1* promoter and fused this fragment with the EGFP reporter (*N-box*:*EGFP*). This construct was injected into zebrafish embryos, which resulted in scattered cells with green florescent signals, indicating a successful transient expression of the EGFP reporter. These EGFP-positive cells were overlapped with HuC/D-positive neurons, indicating that they are neurons in which EGFP is faithfully driven by the regulatory element containing the N-boxes (Additional file 2). Co-injection of *N-box*:*EGFP* with full length *her2* or *her8a* cRNA downregulated the expression of EGFP in the HuC/D-positive neurons, whereas co-injection of a *her2* or *her8a* construct lacking the transcription activating WRPW domain [22] did not affect EGFP expression (Additional file 2). This result demonstrated that Her2 and Her8a specifically bind to the N-boxes of *dtx1* in developing neurons.

### Role of Dtx1 in neuronal differentiation

**Fig. 4:**
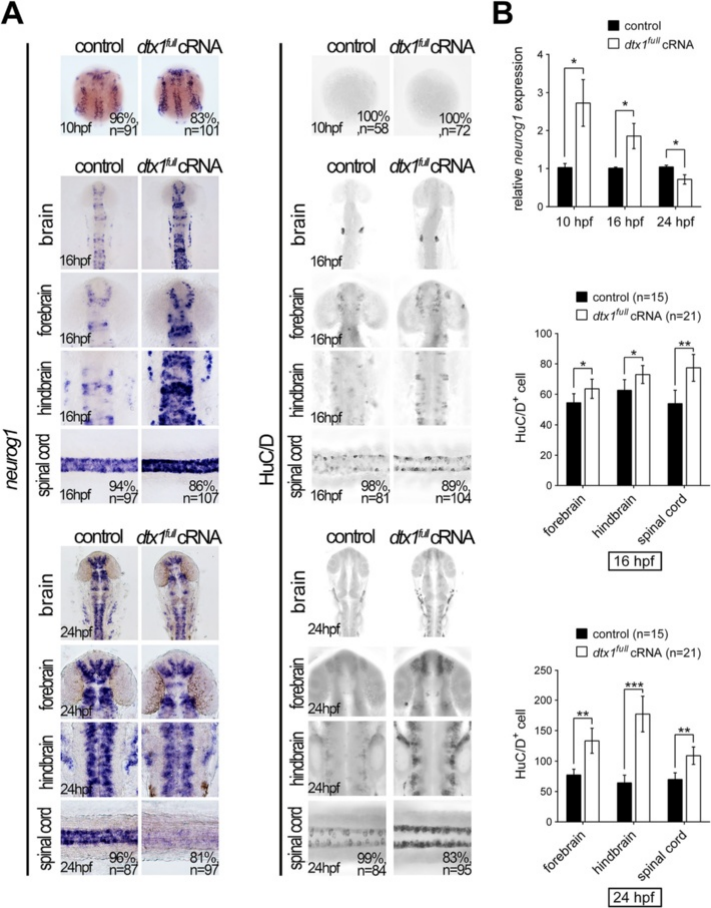
Dtx1 induces the premature differentiation of neuronal precursors. Embryos were examined through *in situ* hybridization by using *neurogenin1* and immunohistochemistry by using an anti-HuC/D antibody. **a** Compared with the controls, *neurogenin1* expression level was increased at the bud stage but downregulated in *dtx1* ^*full*^-injected embryos. By contrast, a significant upregulation of HuC/D signals was detected in *dtx1* ^*full*^ cRNA-injected embryos. **b** The results in **a** were confirmed using qPCR and cell count. Quantitative data are presented as mean ± standard deviation normalized to the number of controls. *, *P* < 0.05; **, *P* < 0.01

In the developing nervous system, Hairy/E(Spl) homologs can suppress neurogenesis in a Notch-dependent manner [26, 27], and a defect in Notch signaling causes a loss of inhibitory effect on neuronal differentiation and consequently results in the precocious generation of early differentiating neurons [20, 28]. Because we observed upregulated *dtx1* expression in Delta–Notch- and Hairy/E(Spl)-deficient embryos, we examined the role of Dtx1 in neural development by overexpressing an *in vitro*-synthesized complete coding sequence of *dtx1* cRNA (*dtx1*^*full*^). We first analyzed the effect of *dtx1*^*full*^ overexpression in the developing nervous system by examining *neurogenin1*-positive neuronal precursors during early neurulation. Whole-mount *in situ* hybridization showed that *neurogenin1* expression was increased in the *dtx1*^*full*^-injected embryos at the bud stage (Fig. 4a). qPCR analysis confirmed a 2.8-fold increase in *neurogenin1* expression in *dtx1*^*full*^-injected embryos (Fig. 4b). These results indicated that Dtx1 was sufficient for inducing the formation of neuronal precursors. We investigated whether the increased number of neuronal precursors after *dtx1*^*full*^ overexpression was due to the elicited premature neuronal differentiation observed in Notch-deficient embryos. Immunohistochemistry analysis conducted using the postmitotic neuronal marker HuC/D antibody revealed considerable HuC/D upregulation in *dtx1*^*full*^-injected embryos from 14 hpf onwards (Fig. 4). This effect was confirmed by Western blotting that showed a 1.5 to 2-fold increase in HuC/D expression in *dtx1*^*full*^-injected embryos (Fig. 4). Concurrently with increased HuC/D expression, *neurogenin1* expression was initially upregulated at the bud stage but later downregulated at 24 hpf (Fig. 4), indicating that neurons were prematurely differentiated from *neurogenein1*-positive precursors into HuC/D-positive differentiating neurons (Fig. 4). These results suggested that *dtx1*^*full*^ cRNA injection was necessary for eliciting premature neuronal differentiation.

Notch signaling also plays a role in cell proliferation and survival [1, 29]. Therefore, we examined whether Dtx1 also regulates neural cell proliferation and apoptosis. Proliferating neurons were analyzed using a phospho-histone H3 antibody and counterstained with *neurogenin1*. The results showed that neural proliferation remained unchanged after *dtx1*^*full*^ injection (Additional file 3), indicating that Dtx1 has no effect on neuronal proliferation. We tested for neuronal apoptosis by using immunohistochemistry to detect the presence of activated caspase-3 counterstained with *neurogenin1*. No significant differences were observed between *dtx1*^*full*^ overexpression and controls (Additional file 3), suggesting that Dtx1 does not play a role in neuronal precursor cell apoptosis. Our results demonstrated that Dtx1 induced neuronal differentiation without affecting cell proliferation and apoptosis.

### Role of Dtx1 deficiency in defective neurogenesis

**Fig. 5:**
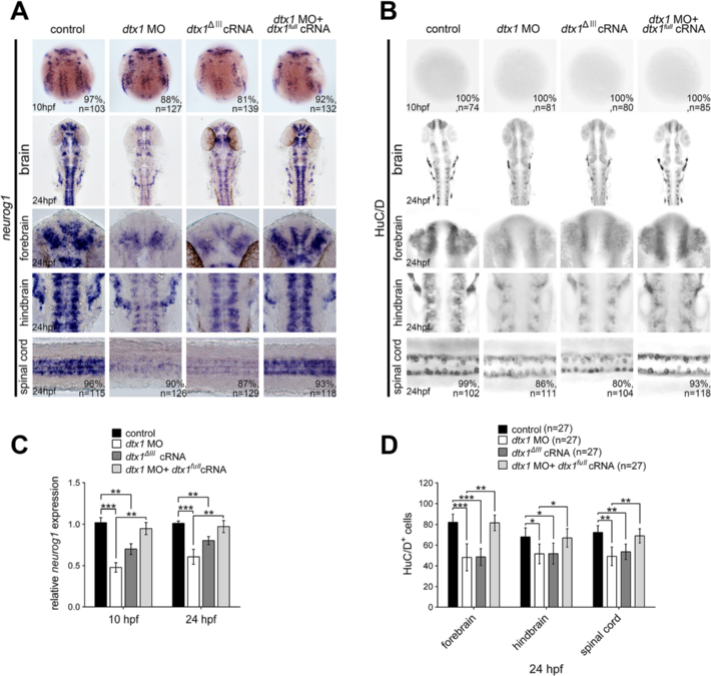
Disruption of Dtx1 expression by using *dtx1* ^*ΔIII*^ or *dtx1* morpholino reduces neuronal differentiation. **a** The injection of *dtx1* ^*ΔIII*^ or *dtx1* morpholino caused an identical phenotype, which downregulated *neurog1* (**a**) and HuC/D expression (**b**) The phenotypes caused by the morpholino injection could be rescued by a concomitant injection of *dtx1* ^*full*^ cRNA. The embryo stages are shown in the bottom left corner of each panel. **c** qPCR analysis confirmed the results obtained through *in situ* hybridization in **a d** Counting the HuC/D-positive cells confirmed the results obtained in **b** *, *P* < 0.05; **, *P* < 0.01; ***, *P* < 0.001

Studies have shown that the RING finger motif is essential for Deltex function during *Drosophila* wing formation and rat oligodendrocyte development [11, 30]. Accordingly, we created a deletion zebrafish *dtx1* cDNA construct lacking the RING domain (*dtx1*^*ΔIII*^) to gain insights into the structural requirements for Dtx1 function. We found that *neurogenin1* was downregulated in the *dtx1*^*ΔIII*^-injected embryos at the bud stage and 24-hpf embryos (Fig. 5). HuC/D expression analysis also revealed downregulation in *dtx1*^*ΔIII*^-injected embryos (Fig. 5). This result suggested that *dtx1*^*ΔIII*^ was effective in disrupting neuronal differentiation.

The injection of *dtx1*^*full*^ and *dtx1*^*ΔIII*^ caused opposite effects on neuronal differentiation, suggesting that *dtx1*^*ΔIII*^ acts as a dominant-negative construct. To further confirm that *dtx1*^*ΔIII*^ can have a dominant-negative effect and examine the physiological role of Dtx1, we blocked Dtx1 protein production by using the morpholino-knockdown approach. Two 25-bp antisense morpholinos (MO1 and MO2) were synthesized to target different regions located adjacent to the translation start site of *dtx1* mRNA. To confirm the efficacy of the approach, each of the two *dtx1* morpholinos was coinjected with cRNA of a reporter construct that contained the *dtx1* MO1 and MO2 binding sequences upstream of an EGFP reporter (5′*dtx1-EGFP*). Effective knockdown, as revealed by the loss of the EGFP protein, was observed after coinjection with either of the two *dtx1* morpholinos, whereas no reduction in EGFP protein expression was observed after coinjection with a control morpholino (Additional file 4). Injecting 2.5 ng of MO1 or 5.0 ng of MO2 resulted in identical phenotypes; therefore, only embryos injected with 2.5 ng of MO1 are shown. Injecting *dtx1* morpholinos downregulated the expression of the neuronal precursor gene *neurogenin1* and postmitotic neuron marker HuC/D (Fig. 5) and this phenotype persisted until the last stage examined (Additional file 5), indicating that Dtx1 was essential for neuron formation. Morpholino specificity was confirmed using rescue experiments, in which the morpholinos were coinjected with *dtx1*^*full*^ cRNA or human *DTX1* cRNA (Fig. 5 and Additional file 6). The phenotypes caused by Dtx1 knockdown were identical to those observed in *dtx1*^*ΔIII*^-injected embryos. These results indicated that the RING finger domain is essential for Dtx1 function and that the deletion of this domain causes a dominant-negative effect. These results also confirmed the specificity of the phenotypes conferred by the *dtx1* morpholino injection. Functional disruption by using *dtx1*^*ΔIII*^ or *dtx1* morpholinos did not affect the proliferation and apoptosis of neurons (Additional file 3), confirming that Dtx1 is nonessential for neuronal proliferation and survival.

Studies have shown that morpholinos can cause off-target apoptosis mediated by p53 activation (Robu et al. 2007). To exclude this possibility, all the *dtx1* morpholinos were coinjected with a *tp53* MO. The results revealed no significant differences between the phenotypes of embryos coinjected with *dtx1* and *tp53* MOs and those injected with *dtx1* MO alone (Additional file 7). Because the phenotypes resulting from the *dtx1* morpholino injection were also rescued by the concomitant injection of *dtx1* cRNA and no abnormal cell apoptosis occurred in *dtx1* morphants (as stated previously), the phenotypes of the *dtx1* morphants did not result from p53 activation but rather from the specific inhibition of Dtx1 function.

### Role of Dtx1 in glial differentiation

**Fig. 6:**
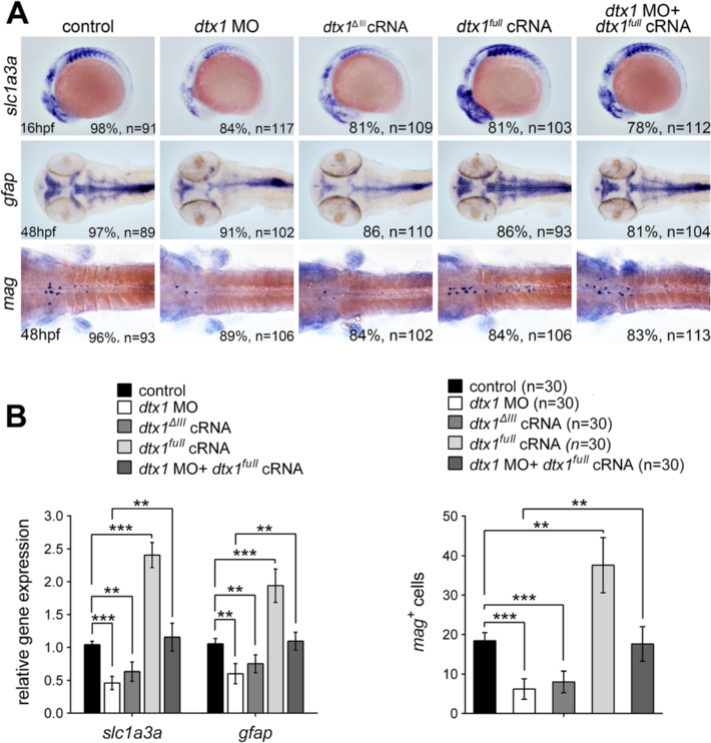
Dtx1 induces the expression of markers for glial precursors and mature glial cells. **a** Expression of *slc1a3a*, *mag*, and *gfap* was induced in embryos that overexpressed *dtx1* ^*full*^. An injection with *dtx1* ^*ΔIII*^ or *dtx1* morpholino downregulated *slc1a3a*, *mag*, and *gfap* expression, and this effect could be rescued by a concomitant injection of *dtx1* ^*full*^ cRNA. The embryo stages are shown in the bottom left corner of each panel. **b** qPCR analysis and cell count confirmed the results obtained through *in situ* hybridization shown in A. *, *P* < 0.05; **, *P* < 0.01; ***, *P* < 0.001

Gliogenesis proceeds after neurogenesis. Recent *in vitro* studies have shown that murine DTX1 is capable of promoting gliogenic specification in neural crest cell cultures [31, 32] and is essential for oligodendrocyte development in primary glial cultures [12, 33]. We accordingly analyzed the role of zebrafish Dtx1 during gliogenesis. Overexpression of *dtx1*^*full*^ cRNA upregulates the expression of the early glial marker *slc1a3a* [*Glast* in mammals, for glial progenitors [23, 34, 35]], increasing the expression 2.5-fold, according to qPCR analysis (Fig. 6). This suggests that Dtx1 is effective in inducing gliogenesis. By contrast, *slc1a3a* was downregulated in embryos injected with *dtx1*^*ΔIII*^ or *dtx1* morpholinos (Fig. 6), and this finding was also confirmed by qPCR analysis showing 2-fold and 1.9-fold decreases (Fig. 6). The effect of *dtx1* morpholinos could be restored by coinjection with *dtx1*^*full*^ cRNA (Fig. 6). To test whether the effects of *slc1a3a* were caused by irregular glial differentiation, we evaluated the expression of the mature glial cell marker myelin-associated glycoprotein (*mag*, for myelinated glial cells) and glial fibrillary acidic protein (*gfap*, for radial glia and astrocytes) [36]. Overexpression of *dtx1* caused increased *mag* expression (2.0-fold increases according to qPCR results; Fig. 6), whereas injecting *dtx1*^*ΔIII*^ or the *dtx1* morpholinos caused decreased *mag* expression (Fig. 6), as confirmed using qPCR (Fig. 6). Overall, these results show that Dtx1 is necessary for inducing glial differentiation.

### Role of Dtx1 and Notch interaction in neuronal and glial differentiation

**Fig. 7:**
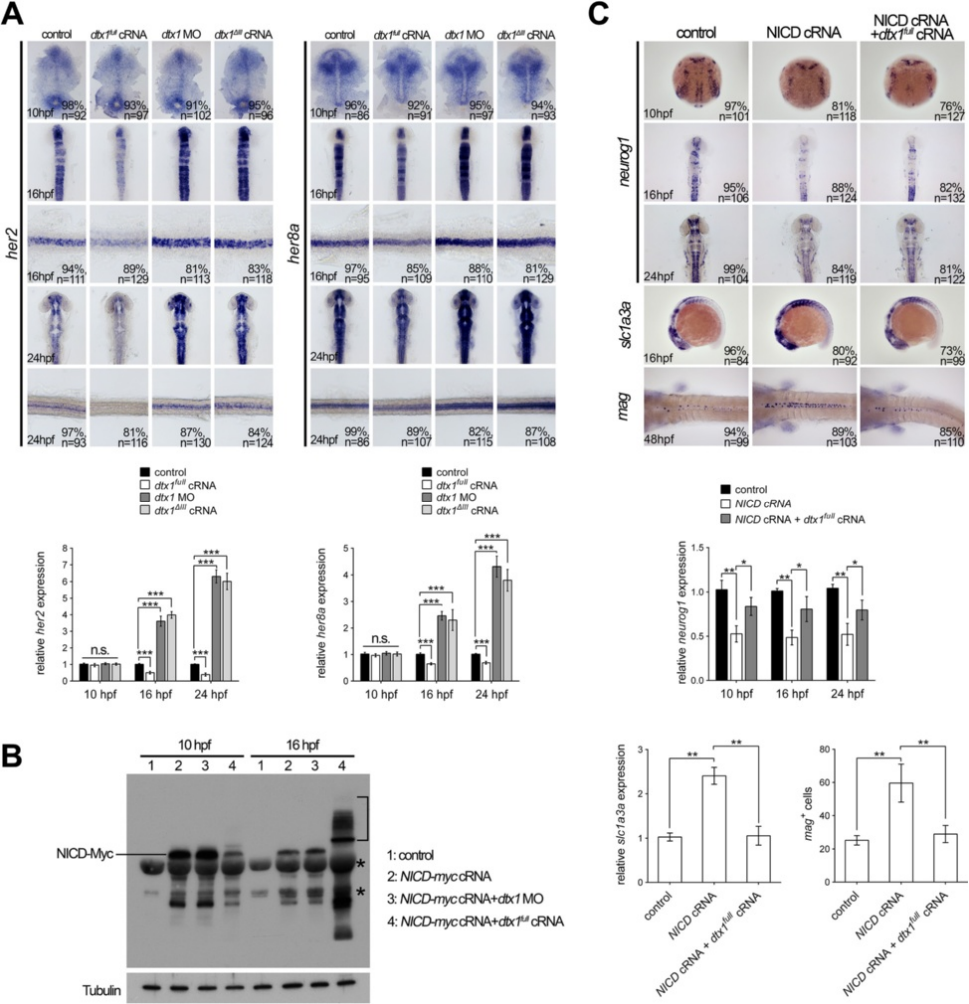
Dtx1 regulates Notch signaling in a temporal manner. **a** *In situ* hybridization and qPCR results show that expression of *her2* and *her8a* was not affected by the alteration of *dtx1* expression at 10 hpf. In contrast, the expression of *her2* and *her8a* was downregulated by *dtx1* ^*full*^ cRNA and upregulated by *dtx1* ^*ΔIII*^ or *dtx1* morpholino after 16 hpf. The embryo stages are shown in the bottom left corner of each panel. **b** Injection of *NICD* inhibits *neurog1* expression and induces *slc1a3a* and *mag* expression. This effect of *NICD* could be abrogated by co-injection with *dtx1* ^*full*^ cRNA at all stage examined. The result of *in situ* hybridization was confirmed by qPCR quantification showing in **a** and **b c** Western blot analysis with anti-Myc antibody revealed that injection of *dtx1* ^*full*^ cRNA caused ubiquitination of NICD-myc, indicated by multiple bands in samples at 16 hpf (bracket). Asterisks indicating non-specific bands

Dtx1 has been identified as a positive and negative mediator for Notch signaling [9, 12, 37]. To examine whether Dtx1 reciprocally regulates Notch signaling in neuronal differentiation, we examined the expression of *her2* and *her8a* in embryos with *dtx1* gain- and loss-of function at different developmental stages. Overexpressing *dtx1*^*full*^ cRNA did not alter the expression of *her2* and *her8a* at 10 hpf, and injection of *dtx1*^*ΔIII*^ or morpholino also had no effect on *her2* and *her8a* expression at 10 hpf (Fig. 7a), indicating that Dtx1 does not reciprocally regulate Notch and HES/Her expression at this stage. Because Dtx1 is regulated by Her2 and Her8a and induces neuronal differentiation at this stage, this result also suggested that Dtx1-mediated regulation of neuronal differentiation does not require a reciprocal regulatory effect on Notch. On the contrary, overexpression of *dtx1*^*full*^ inhibited *her2* and *her8a* expression from 16 hpf, and injection of *dtx1*^*ΔIII*^ or *dtx1* morpholino resulted in upregulation of *her2* and *her8a* after 16 hpf (Fig. 7a), suggesting that Dtx1 inhibits Notch signaling and confirmed that the RING domain is essential for inhibiting Notch signaling in these later stages.

To further examine the effect of Dtx1 on Notch, we tagged the intracellular domain of Notch (NICD) with Myc-tag (*NICD-myc*), and co-injected *NICD-myc* with or without *dtx1*. The result of western blot analysis using anti-Myc antibody showed multiple ladder-like bands that migrated slower than Myc in the sample co-injected with *dtx1*. In comparison, only a single band was observed in the sample without *dtx1* (Fig. 7b), indicating Dtx1-mediated ubiquitination of NICD. However, ubiquitination of NICD was only observed after 16 hpf but not at 10 hpf (Fig. 7b). Because we observed Dtx1 regulates neuronal differentiation at 10 hpf, this result suggested Dtx1 regulates neuronal differentiation independently of its action on NICD; however, events later than 16 hpf, such as gliogenesis, involves the ubiquitination of NICD by Dtx1.

To further examine the role of Dtx1 and Notch interaction in neuronal and glial differentiation, we injected the constitutively active form of Notch, the intracellular domain of Notch (*NICD*). The result showed NICD inhibited the neuron precursor marker *neurog1*, and co-injection of *dtx1*^*full*^ cRNA with *NICD* rescued the expression of *neurog1* (Fig. 7c). Considering that NICD induces *Hes*/*her* expression, along with our finding that Her2 and Her8a inhibits *dtx1* expression, the rescue effect of *dtx1* for NICD injection confirmed that Dtx1 acts downstream of NICD-Her signaling. However, with respect to Notch regulation by Dtx1, we think this result did not provide a substantial evidence to show that Dtx1 acts on NICD in neuronal differentiation. Indeed, we found that Dtx1 does not regulate the expression of *her2* and *her8a* at 10 hpf, which supports the idea that Dtx1 does not act on Notch signaling at 10 hpf in neuronal differentiation. In gliogenesis, *NICD* injection induced glial markers *glast* and *mag*, and co-injection of *dtx1*^*full*^ cRNA with *NICD* rescued the expression of glial markers, and at 16 hpf and onwards, Dtx1 was able to cause ubiquitination of NICD and downregulate Notch target genes *her2* and *her8a*. These results suggested negative reciprocal regulation of Dtx1 and Notch-HES/Her in gliogenesis.

## Discussion

Notch signaling plays a fundamental role in various neural developmental processes, such as neuronal progenitor maintenance, and governs the decision between neuronal and glial lineages. Notch activation is also directly implicated in tumor growth through the regulation of cell survival, proliferation, and metastasis [38]. The mechanism by which Notch signaling governs different cellular processes remains unclear. We examined the role of Dtx1 during neural development and obtained the first *in vivo* evidence of Dtx1 as a major element in Notch-mediated neural differentiation. We first focused on the differentiating neurons and found that Notch signaling negatively regulated *dxt1* expression through a direct binding of Hairy/E(Spl) proteins to the promoter region of *dtx1*. This result suggested that Dtx1 is a positive regulator of neuronal differentiation. Dtx1 overexpression induced neuronal differentiation, whereas Dtx1 deficiency showed decreased neuronal differentiation. Therefore, our study revealed Dtx1 as a novel downstream effector of Notch–Hairy/E(Spl)-mediated neuronal differentiation, and this mechanism is specific in neuronal differentiation, but does not affect proliferation and apoptosis. This study therefore described a regulatory mechanism explaining how Notch signaling selectively regulates neuronal differentiation.

Previous studies showed that Deltex can act as either a positive or a negative regulator on Notch signaling depending on the developmental and cellular context [6–8]. In addition, whether this regulation is essential for the physiological role of Deltex proteins is not clear. In the present study, we showed that during the early stage, the expression of *dtx1* is regulated by Notch-HES/Her, and Dtx1 induces neuronal differentiation, which does not involve a reciprocal regulatory effect on Notch. On the contrary, at later stages, either NICD or Dtx1 alone is sufficient to induce glial differentiation. This result is supported by previous studies showing that Notch–Hairy/E(Spl) signaling induces glial differentiation [39, 40]. Furthermore, *in vitro* analysis have shown that murine DTX1 is capable of promoting gliogenic specification in neural crest cell cultures [31, 32], and that DTX1 is essential for oligodendrocyte development in primary glial cultures [12, 33]. This result suggested that Dxt1 may function directly on gliogenic factors to induce glial differentiation. In addition, we revealed a novel mechanism in which Dtx1 and Notch inhibit each other via reciprocal negative regulation. Taken together, our results indicate that the negative reciprocal regulation between Dtx1 and Notch provides the proper balance between Dtx1 and Notch expression, which is essential for the fine-tuning of glial differentiation.

Aberrant expression of Notch signaling is often observed in various types of tumor. Studies have suggested that Notch signaling in tumorigenesis is oncogenic and tumor-suppressive, whereas its role in mediating the proliferation and inhibition of differentiation of stem cells may be essential for tumorigenesis [1, 29]. Although the Dtx family is one of the major regulators of Notch signaling, few studies have reported the role of Dtx in tumorigenesis. The role of Dtx proteins in tumorigenesis has recently been noted in lymphoma [7] and osteosarcoma cells [41]. By contrast, based on an analysis of the Cancer Genome Anatomy Project and Gene Expression Omnibus expression databases, we found that DTX1 is expressed at low levels in brain tumors. We analyzed DTX1 expression in several types of brain tumor cell line (T98G, U-87 MG, A172, Hs 683, D341 Med, M059K, CHP-212, and H4), which displayed low DTX1 expression levels (unpublished observation). On the basis of the results of our *in vivo* study of the zebrafish developing nervous system showing that Dtx1 induces neural differentiation and is not involved in proliferation and apoptosis, we suggest that Dtx1/DTX1 does not play an inductive role in the progression of at least some brain tumors. However, a study showed that DTX1 is highly expressed in many glioma samples and glioma cell lines and is necessary for inducing glioma cell proliferation [42]. In addition, a study of the neuroepithelial cell line MNS-70 showed that mouse DTX1 mediates Notch signaling to block neural progenitor cell differentiation [11]. Therefore, the role of Dtx1/DTX1 in tumorigenesis is highly dependent on the cell types, and therefore, the oncogenic role of Dtx1/DTX1 remains inconclusive and must be further examined.

## Conclusions

Although Deltex proteins are major mediators in Notch signaling, their role in development and tumorigenesis remains unclear. We isolated zebrafish Dtx1 and showed that it induces neuronal and glial differentiation without affecting cell proliferation and apoptosis. In addition, *dtx1* transcription was inhibited by the Notch downstream transcription factors Her2 and Her8a. Our result also suggested that Dtx1 acts directly on neuronogenic and gliogenic factors to induce neuronal and glial differentiation, respectively, and that this function does not require a regulatory effect on the Notch-HES/Her pathway. However, Dtx1 and the Notch-HES/Her cascade inhibit each other to achieve the proper balance for glial differentiation. Therefore, Dtx1 interacts differently with Notch–Hairy/E(Spl) in different cell types. In conclusion, our results provide information for a clearer understanding of the role of Dtx1 and the mechanism underlying Notch signaling in neural development and the progression of brain tumors.

## Methods

### Ethics statement

All experiments were performed in strict accordance with the standard guidelines for zebrafish work and approved by the Institutional Animal Care and Use Committee of Chang Gung University (IACUC approval numbers: CGU08-86 and CGU11-118).

### Fish maintenance and mutants

Tü (wild-type) zebrafish embryos were purchased from the Zebrafish International Resource Center (Eugene, OR, USA) and were raised, maintained, and paired under standard conditions. The embryos were staged according to somite numbers, hours postfertilization (hpf), and days postfertilization (dpf) [43].

### Sequence comparisons

Amino acid sequences were aligned and displayed using Vector NTI (Invitrogen). The GenBank accession numbers of the compared proteins are as follows: Drosophila Deltex (NM_078509), zebrafish Dtx1 (KR869089), mouse DTX1 (NM_008052), mouse DTX2 (NM_001256096), mouse DTX3 (NM_030714), mouse DTX4 (NM_172442), human DTX1 (NM_004416), human DTX2 (NM_020892), human DTX3 (NM_178502), and human DTX4 (NM_015177).

### Construct generation

Human DTX1 was used as a template to screen the zebrafish genome database (www.ensembl.org, version Zv8), and a fragment with high similarity was identified (ENSDART00000091274). Sequence comparison with mammalian homologs showed that this fragment was not a complete coding region. The missing 5′- and 3′-end sequences were then amplified by rapid amplification of cDNA ends (RACE) using the 5′-RACE and 3′-RACE kits (Ambion) according to the manufacturer’s instructions. The open-reading frame of zebrafish *dtx1* (*dtx1*^*full*^) was polymerase chain reaction (PCR)-amplified with high-fidelity Pfu polymerase (Fermentas) and primers (5′-CGGGATCCCGGCCACCATGGCTCGACCCGGAGCGCTG-3′ and 5′- GCTCTAGAGCCCATTCGTCTTTCAGGTTGTCCTC-3′). *dtx1*^*ΔIII*^ was created with primers 5′- CGGGATCCCGGCCACCATGGCTCGACCCGGAGCGCTG-3′ and 5′-GCTTTCTGGAGCACACTTGATCTTCTCTGTG-3′. *Dtx1* MO1 and MO2 binding sequences were inserted upstream of an enhanced green fluorescent protein (EGFP) reporter in the pCS2 vector to create a *5*′*dtx1-EGFP* construct for evaluating the specificity and efficiency of morpholinos. The open-reading frame of human *DTX1* was amplified by PCR using the primers 5′- GAATTCGCCACCATGTCACGGCCAGGCCACG-3′ and 5′- GAATTCAGCCTTGGCTGCAGCCTCGTGA-3′. The 2849 bp fragment containing the N-boxes in the *dtx1* promoter (*N-box:EGFP*) was PCR-amplified using the primers 5′-ATCGATCTCCACGTCTCTCCAGTGTGACCTTC-3′ and 5′-GGATCCTGACCCAGCTCACAGCTGGGTTATC-3′. The carboxyl terminus was fused with EGFP and inserted into a Tol2 vector [44]. The open-reading frame and WRPR deletion constructs of *her2* and *her8a* were described previously [22, 23].

### RNA and morpholino injection

Capped RNA encoding the complete coding sequences of *dtx1* and *dtx1*^*ΔIII*^ was prepared as described previously [23]. Antisense morpholino oligonucleotides were purchased from Gene Tools, LLC (Philomath, OR, USA). Two morpholinos that targeted the 5′-end to the ATG start codon of *dtx1* (MO1: −45 to −21, TTATCGACCCAGCTCACACAAGGGC and MO2: −75 to −51, ACACACCAGCGAACGTCTTCCCAAT) were used, resulting in the same phenotype. Basic Local Alignment Search Tool analysis revealed homology of less than 20-bp identity for MO1 or MO2 to other genomic sequences, none of which corresponded to the 5′-UTR or exon–intron splicing site of predicted or characterized genes, suggesting that MO1 and MO2 act specifically on *dtx1*. A control morpholino, designed according to a random nucleotide sequence not found in the zebrafish genome (5′-CCTCTTACCTCAGTTACAATTTATA-3′; Gene Tools), and a morpholino with 5 bases mismatched to MO1 (5′-TTATaGACaCAGaTCAtACAAaGGC-3′; small letters indicate mismatched bases) were injected in an equal amount of MO1 as a control experiment. All injections were performed at the one- or two-cell stage, and cRNAs or morpholinos were introduced into blastomeres. For rescue experiments, embryos were first injected with the *dtx1* morpholino, and then with the *dtx1* cRNA by using a separate needle to ensure that the morpholino was effective.

### Chemical treatment

Notch signaling was inactivated by DAPT (EMD Millipore) treatment. DAPT was dissolved in dimethyl sulfoxide (DMSO) to prepare a 10-mM stock solution. The stock solution was added to the embryos to reach a final concentration of 100 μM DAPT. The control embryos were treated with an identical amount of DMSO.

### Histological analysis

Digoxigenin-UTP-labeled riboprobes were synthesized according to the manufacturer’s instructions (Roche), and *in situ* hybridizations were performed as described previously [45]. The color reaction was conducted using the NBT/BCIP substrate (Roche). To minimize the variation between the control and experimental groups, we used embryos produced by a single pair of parents and always used the same number of embryos for the control and experimental groups. The groups were compared under precisely same experimental conditions at the same time, and color reactions were initiated and stopped at precisely the same time. For immunohistochemistry, zebrafish embryos were blocked in 5 % goat serum and incubated with a rabbit phospho-histone H3 antibody or rabbit monoclonal antiactive caspase-3 (1:200, Abcam). Goat antimouse IgG HRP or goat antirabbit IgG HRP (Roche) was used for detecting the primary antibodies, and DAB was used as a substrate for the secondary antibody-conjugated HRP (Amresco). The embryos were mounted in Vectashield mounting medium (Vector Laboratories, Inc.).

### Chromatin immunoprecipitation and PCR

The complete coding sequences of *her2* and *her8a* were inserted into a pCS2-MT vector containing six repeats of a Myc tag. Zebrafish embryos at the one-cell stage were injected with *her2*-*myc* or *her8a*-*myc* cRNA for ChIP analysis. ChIP was performed using 75 % epiboly zebrafish embryos according to previously described methods [46] by using an anti-Myc tag antibody (EMD Millipore). In general, the embryos were manually dechorionated and fixed with formaldehyde. The unreacted formaldehyde was quenched using glycine, and the chromatin was isolated and precipitated using a Magna ChIP A/G Chromatin Immunoprecipitation Kit (EMD Millipore). The isolated chromatin was sonicated to an average size of approximately 300 bp. Protein A/G magnetic beads were then added to the chromatin solution and incubated overnight at 4 °C with an antibody against Myc (2.5 μg of antibody for each immunoprecipitation experiment). In each ChIP experiment, a portion of the chromatin solution corresponding to 1 % of that used in the ChIP reaction was used as an input DNA control. The protein A/G bead–antibody and chromatin complex was pelleted using a magnetic separator, and the protein/DNA complexes were recovered with an elution buffer. DNA was purified using a polypropylene spin column after immunoprecipitation. Immunoprecipitated DNA and input DNA were used as templates for PCR amplification, and the primers are listed in Additional file 8: Table S1.

### Quantitative analysis

For quantitative real-time PCR (qPCR), embryos were homogenized in the TRIzol reagent (Invitrogen), and total RNA was extracted using a standard method. cDNA was synthesized from total RNA by using random hexamer priming and a RevertAid First Strand cDNA Synthesis Kit (Fermentas). qPCR was performed on an ABI StepOne Real-Time PCR System (Applied Biosystems) with SYBR green fluorescent label (Fermentas). Primers for *dtx1* (F: 5’-AGCATCCAGAACGCTTACGA-3’; R: 5’-GTGCCCGAATTTGTTCCCAC-3’), *neurogenin1* (F: 5’-CGCACACGGATGATGAAGACTCGCG-3’; R: 5’-CGGTTCTTCTTCACGACGTGCACAGTGG-3’), *slc1a3* (F: 5’-GTAACGGGGAGACGCGTCTGCAGCG-3’; R: 5’-GATTATTCCCACGATGACGGCGGCG-3’), *mag* (F: 5’-GTGGATGCCCAGAGACATTT 3’; R: 5’TCCGTCCCTTGTAACTTTCG-3’) and *gapdh* (F: 5’-ACCCGTGCTGCTTTCTTGAC-3’; R: 5’-GACCAGTTTGCCGCCTTCT-3’) were used. Gene expression levels were normalized to *gapdh* expression levels and assessed using the comparative CT method (40 cycles) according to the manufacturer’s instructions (Applied Biosystems).

Statistical analyses were performed using a Student *t* test by using Microsoft Excel 2007. The significance level was set at *P* < 0.05. All reactions were performed in triplicate for each sample.

## Additional files

Additional file 1:
**Alignment of Deltex homologs and synteny comparison.** a. Amino acid alignment of Drosophila, human, mouse, and zebrafish DTX1/Dtx1 sequences. Identical residues across all proteins are marked with black boxes, whereas similar residues are shown by gray boxes. The WWE domain, proline-rich domain, and RING finger motif are indicated. b. The phylogenetic tree of the Deltex protein family. Complete coding protein sequences were used for each family member. Trees were calculated using bootstrapping with 100 replicates. The phylogram shows only the sequence relationships; it does not imply absolute sequence ancestry because no ancestral relationship was assumed in the initial alignments. Genes are not drawn to scale. The initial letter “d” denotes Drosophila, “h” denotes human, “m” denotes mouse, and “z” denotes zebrafish. (TIF 893 kb) Additional file 2:
**Her2 and Her8a specifically bind to the** **N-box** **in** ***dtx1*** **promoter.** a. Green fluorescence indicates EGFP expression driven by the fragment containing N-boxes, which is overlapped with HuC/D-positive neurons (red). b. The intensity of EGFP expression in HuC/D-positive cells, quantified by ImageJ, showing co-injection of *N-box:EGFP* with full-length *her2* or *her8a* cRNA downregulated the expression of EGFP, whereas co-injection of a *her2* or *her8a* construct lacking the transcription activating WRPW domain (*her2* ^*ΔWRPW*^ or *her8a* ^*ΔWRPW*^, respectively) with *N-box:EGFP* does not downregulate EGFP expression. (TIF 997 kb) Additional file 3:
**Disruption of Dtx1 expression had no effects on neuronal proliferation and apoptosis.** **a**. Proliferating neurons were double-labeled with phospho-histone H3 antibody (brown) and *neurogenin1* riboprobes (purple). Apoptotic neuronal precursor cells were labeled for *neurogenin1* (fluorescent green) and activated caspase-3 antibody (fluorescent red). **b**. Proliferating neuronal cells were quantified by counting the proportions of phospho-histone H3- and *neurogenin1-*positive cells, whereas apoptotic neural progenitor cells were quantified by counting the proportions of activated caspase-3- and *neurogenin1-*positive cells, revealing no marked differences between the embryos injected with *dtx1* ^*full*^, *dtx1* morpholinos, and the controls. n.s., nonsignificant. (TIF 2461 kb) Additional file 4:
***dtx1*** **Morpholino specifically and effectively downregulated Dtx1 function.** Embryos injected with the construct containing *dtx1* morpholino-binding sequence fused upstream of *egfp* (*5′dtx1:egfp*) alone or in combination with a control morpholino or a *dtx1* morpholino. Compared with controls, eGFP expression in *dtx1* morphants was downregulated. (TIF 438 kb) Additional file 5:
**Injection of** ***dtx1*** **morpholino downregulates** ***neurog1*** **after 24 hpf.** *neurog1* expression level was downregulated from 48 hpf to 72 hpf in *dtx1* morpholino injected embryos analyzed by *in situ* hybridization (**a**) and qPCR (**b**). *, *P* < 0.05. (TIF 3759 kb) Additional file 6:
**The phenotypes caused by morpholino injection could be rescued by human** ***DTX1*** **cRNA.** Concomitant injection of human *DTX1* cRNA with *dtx1* MO1 rescued the neuronal and glial phenotypes caused by Dtx1 knockdown analyzed by in situ hybridization (**a**) and qPCR (**b**). (TIF 3052 kb) Additional file 7:
**Phenotypes of Dtx1 morphants were not caused by nonspecific p53 activation.** Embryos coinjected with *dtx1* MO and *tp53* MO were compared with Dtx1 morphants, and we observed no detectable deviation from all markers tested. Compared with the controls, the injection of *tp53* MO alone did not show any significant alterations, as shown by *in situ* hybridization (**a**), immunohistochemistry (**b**), qPCR (**c**), and cell count (D).*, *P* < 0.05; **, *P* < 0.01; n.s., nonsignificant. (TIF 2990 kb) Additional file 8: Table S1. Primers used for ChIP-PCR analysis. (TIF 156 kb)

## Abbreviations

ChIP: Chromatin immunoprecipitation
dpf: days post fertilization
Dtx: Deltex
EGFP: Enhanced green fluorescent protein
E(Spl): Enhancer-of-split
hpf: hours post fertilization
MO: Morpholino
qPCR: quantitative real-time PCR
RACE: Rapid amplification of cDNA ends
RING: Really Interesting New Gene finger domain
