# Supplementary figures and images for: Deltex1 is inhibited by the Notch–Hairy/E(Spl) signaling pathway and induces neuronal and glial differentiation
Source: Neural Dev. 2015 Dec 30;10:28. doi: 10.1186/s13064-015-0055-5 (PMC4696291; doi:10.1186/s13064-015-0055-5)

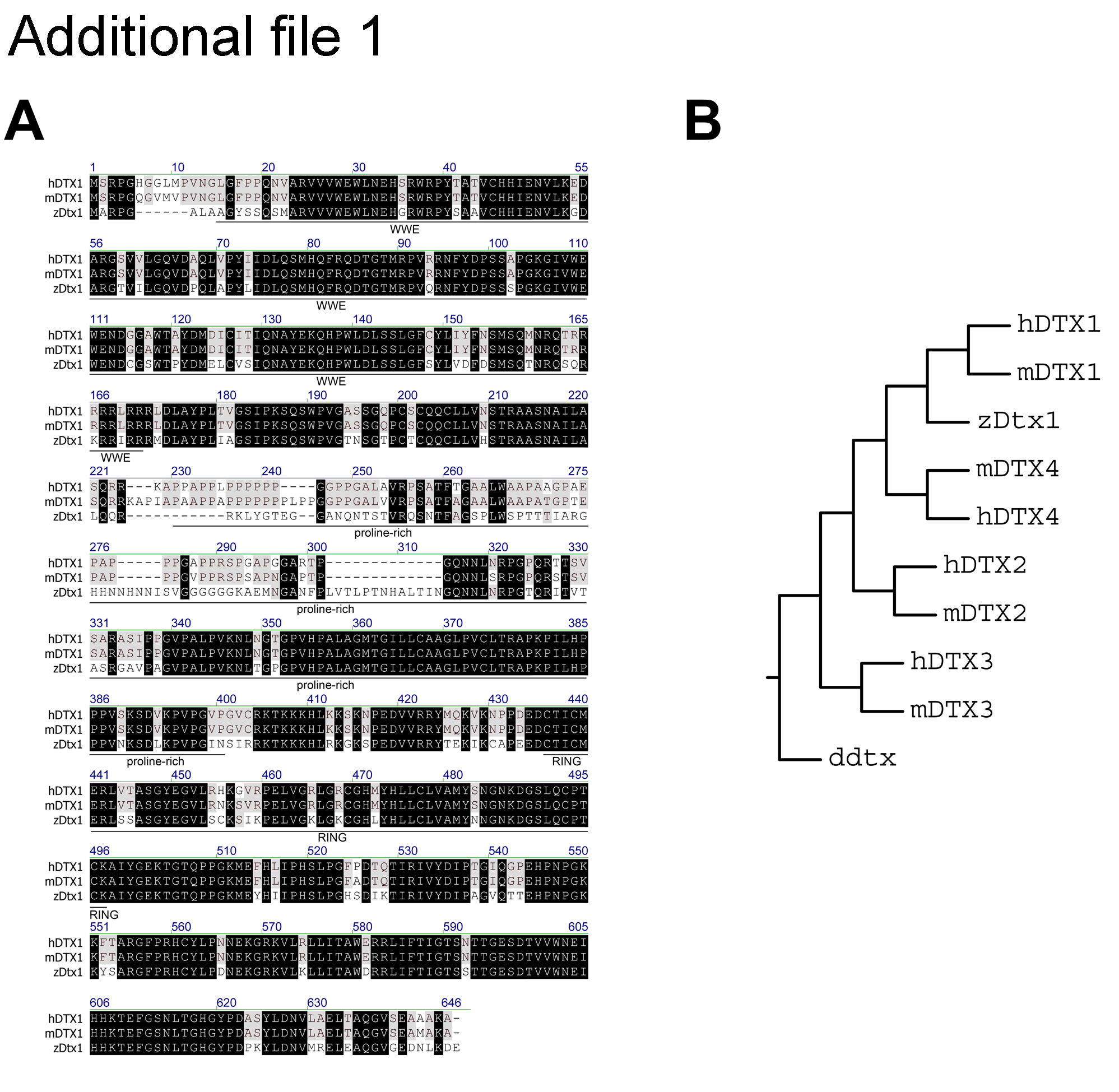

Supplement: Additional file 1: — Alignment of Deltex homologs and synteny comparison. a. Amino acid alignment of Drosophila, human, mouse, and zebrafish DTX1/Dtx1 sequences. Identical residues across all proteins are marked with black boxes, whereas similar residues are shown by gray boxes. The WWE domain, proline-rich domain, and RING finger motif are indicated. b. The phylogenetic tree of the Deltex protein family. Complete coding protein sequences were used for each family member. Trees were calculated using bootstrapping with 100 replicates. The phylogram shows only the sequence relationships; it does not imply absolute sequence ancestry because no ancestral relationship was assumed in the initial alignments. Genes are not drawn to scale. The initial letter “d” denotes Drosophila, “h” denotes human, “m” denotes mouse, and “z” denotes zebrafish. (TIF 893 kb) [file 13064_2015_55_MOESM1_ESM.tif]

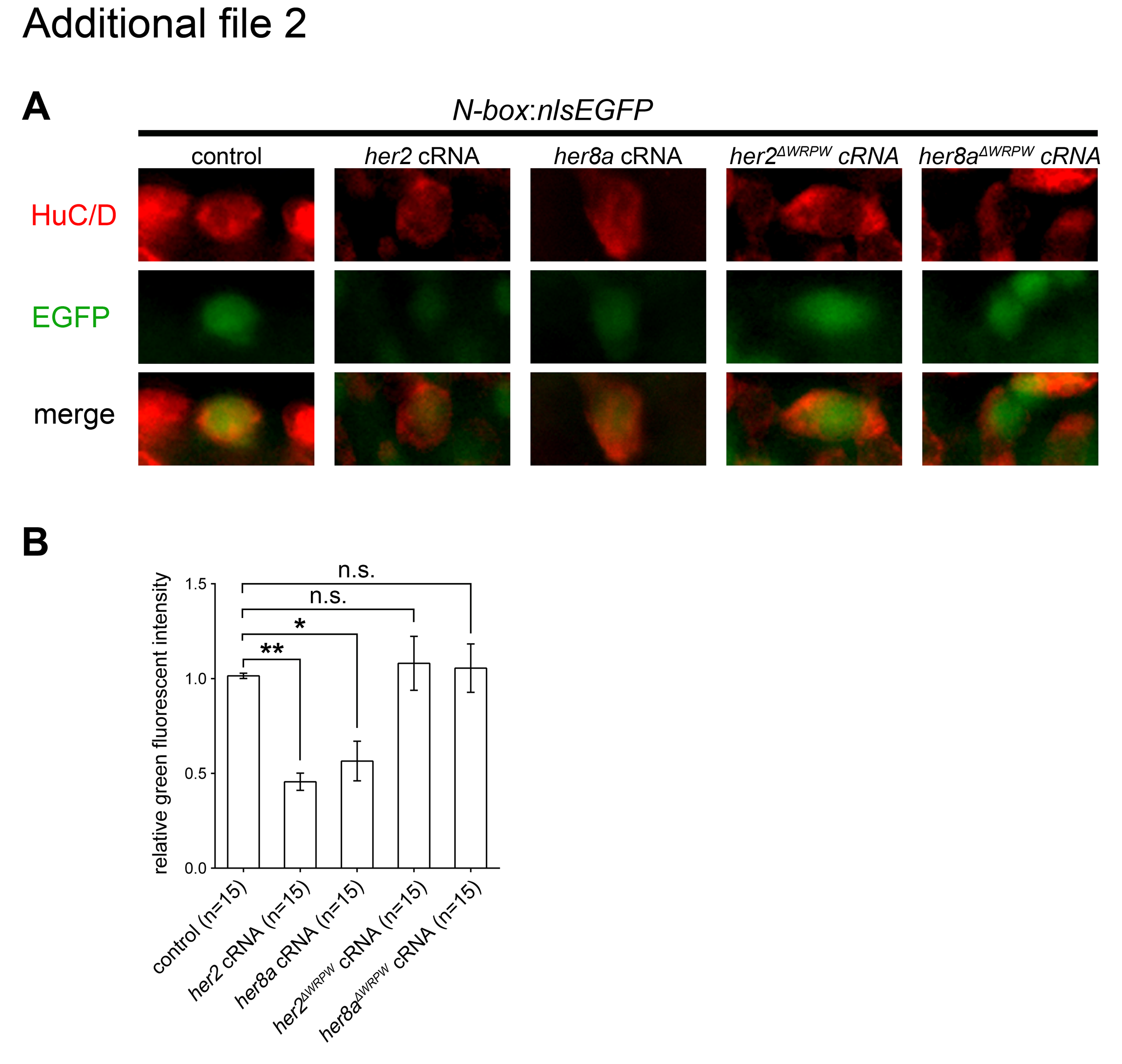

Supplement: Additional file 2: — Her2 and Her8a specifically bind to the N-box in dtx1 promoter. a. Green fluorescence indicates EGFP expression driven by the fragment containing N-boxes, which is overlapped with HuC/D-positive neurons (red). b. The intensity of EGFP expression in HuC/D-positive cells, quantified by ImageJ, showing co-injection of N-box:EGFP with full-length her2 or her8a cRNA downregulated the expression of EGFP, whereas co-injection of a her2 or her8a construct lacking the transcription activating WRPW domain (her2 ΔWRPW or her8a ΔWRPW, respectively) with N-box:EGFP does not downregulate EGFP expression. (TIF 997 kb) [file 13064_2015_55_MOESM2_ESM.tif]

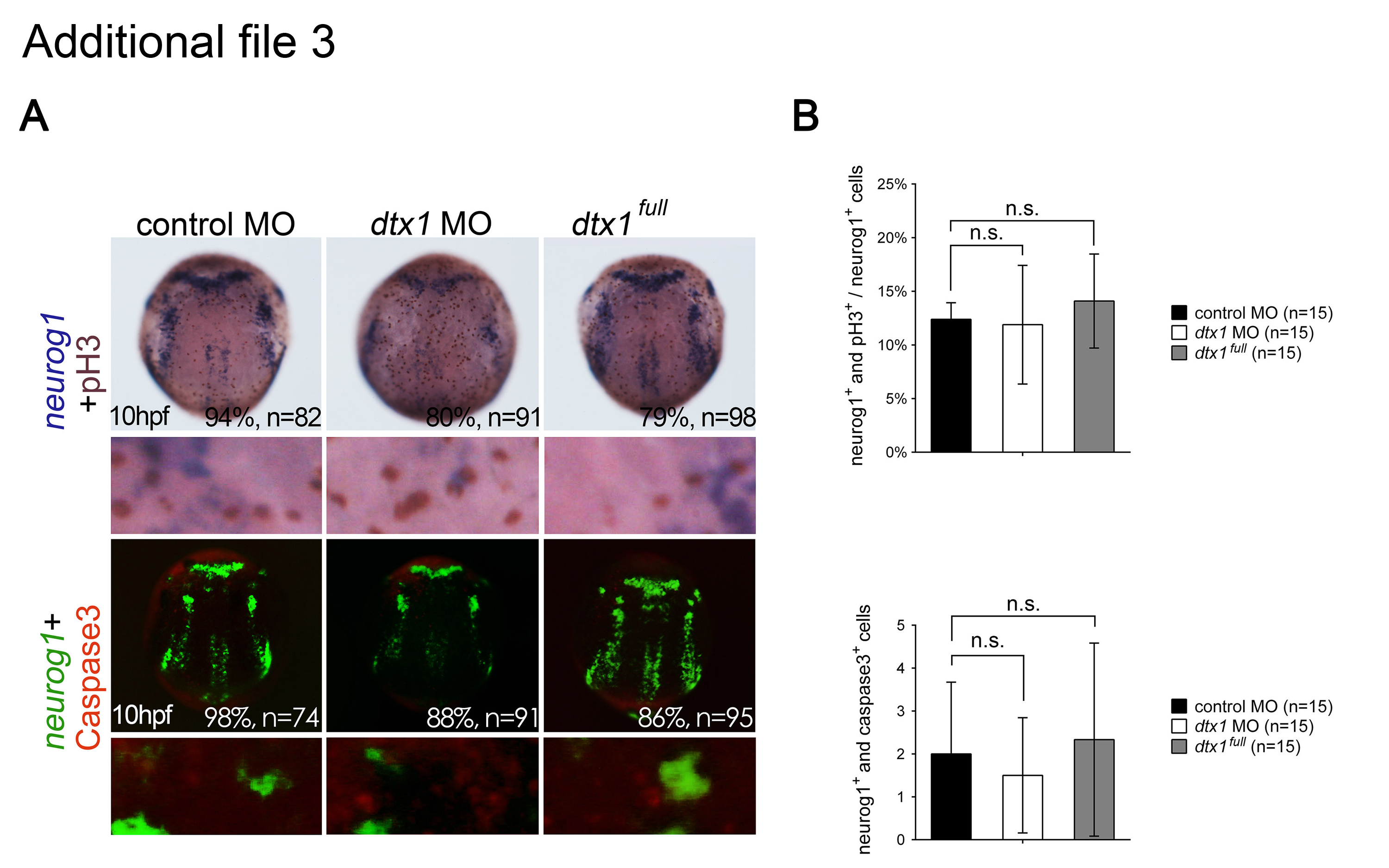

Supplement: Additional file 3: — Disruption of Dtx1 expression had no effects on neuronal proliferation and apoptosis. a. Proliferating neurons were double-labeled with phospho-histone H3 antibody (brown) and neurogenin1 riboprobes (purple). Apoptotic neuronal precursor cells were labeled for neurogenin1 (fluorescent green) and activated caspase-3 antibody (fluorescent red). b. Proliferating neuronal cells were quantified by counting the proportions of phospho-histone H3- and neurogenin1-positive cells, whereas apoptotic neural progenitor cells were quantified by counting the proportions of activated caspase-3- and neurogenin1-positive cells, revealing no marked differences between the embryos injected with dtx1 full, dtx1 morpholinos, and the controls. n.s., nonsignificant. (TIF 2461 kb) [file 13064_2015_55_MOESM3_ESM.tif]

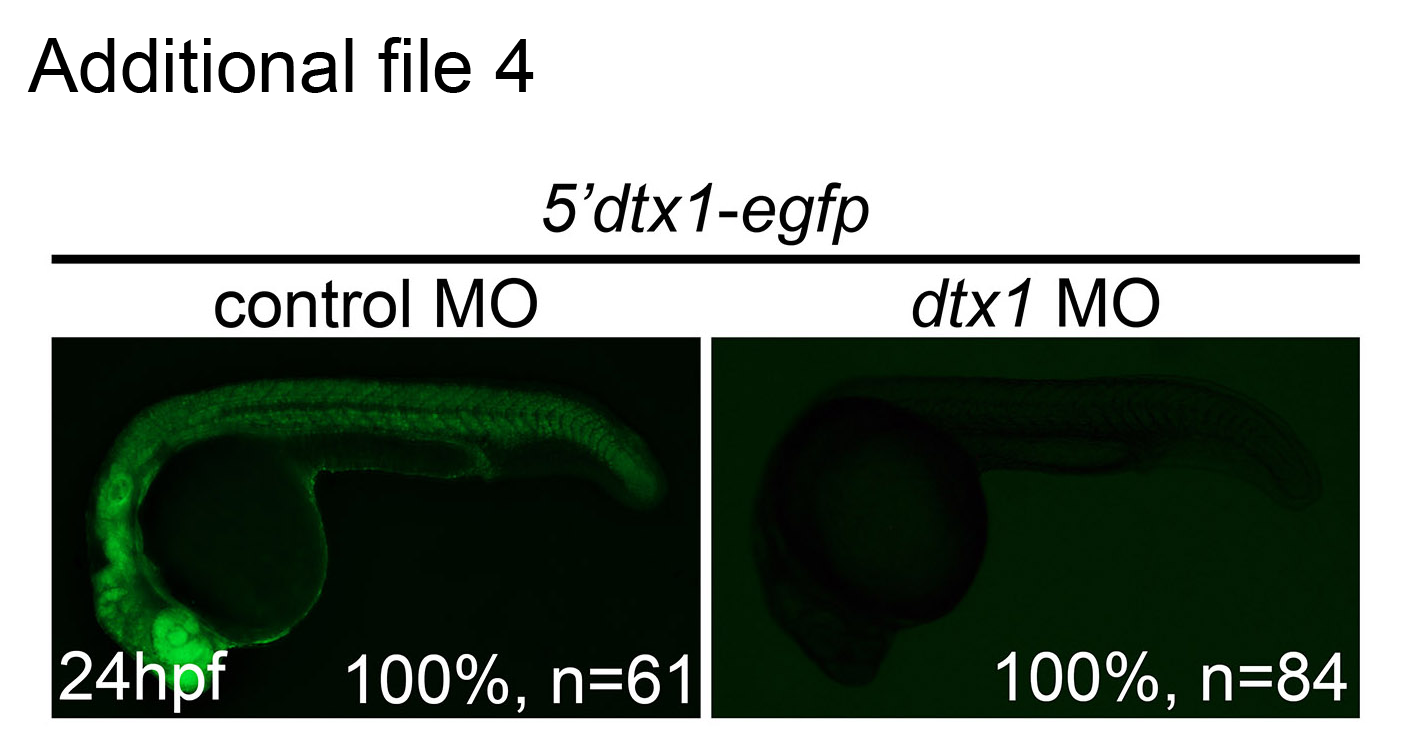

Supplement: Additional file 4: — dtx1 Morpholino specifically and effectively downregulated Dtx1 function. Embryos injected with the construct containing dtx1 morpholino-binding sequence fused upstream of egfp (5′dtx1:egfp) alone or in combination with a control morpholino or a dtx1 morpholino. Compared with controls, eGFP expression in dtx1 morphants was downregulated. (TIF 438 kb) [file 13064_2015_55_MOESM4_ESM.tif]

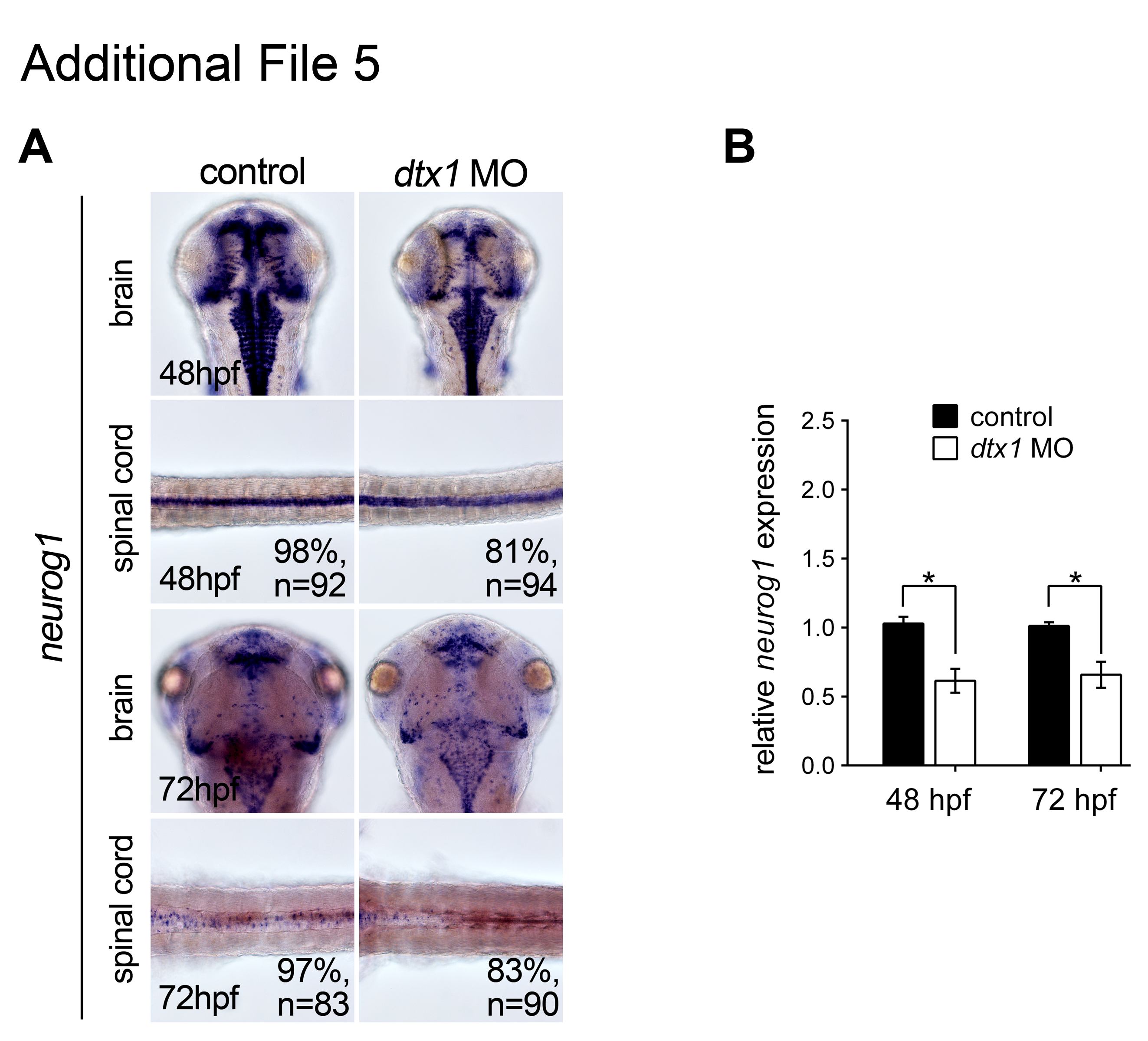

Supplement: Additional file 5: — Injection of dtx1 morpholino downregulates neurog1 after 24 hpf. neurog1 expression level was downregulated from 48 hpf to 72 hpf in dtx1 morpholino injected embryos analyzed by in situ hybridization (a) and qPCR (b). *, P < 0.05. (TIF 3759 kb) [file 13064_2015_55_MOESM5_ESM.tif]

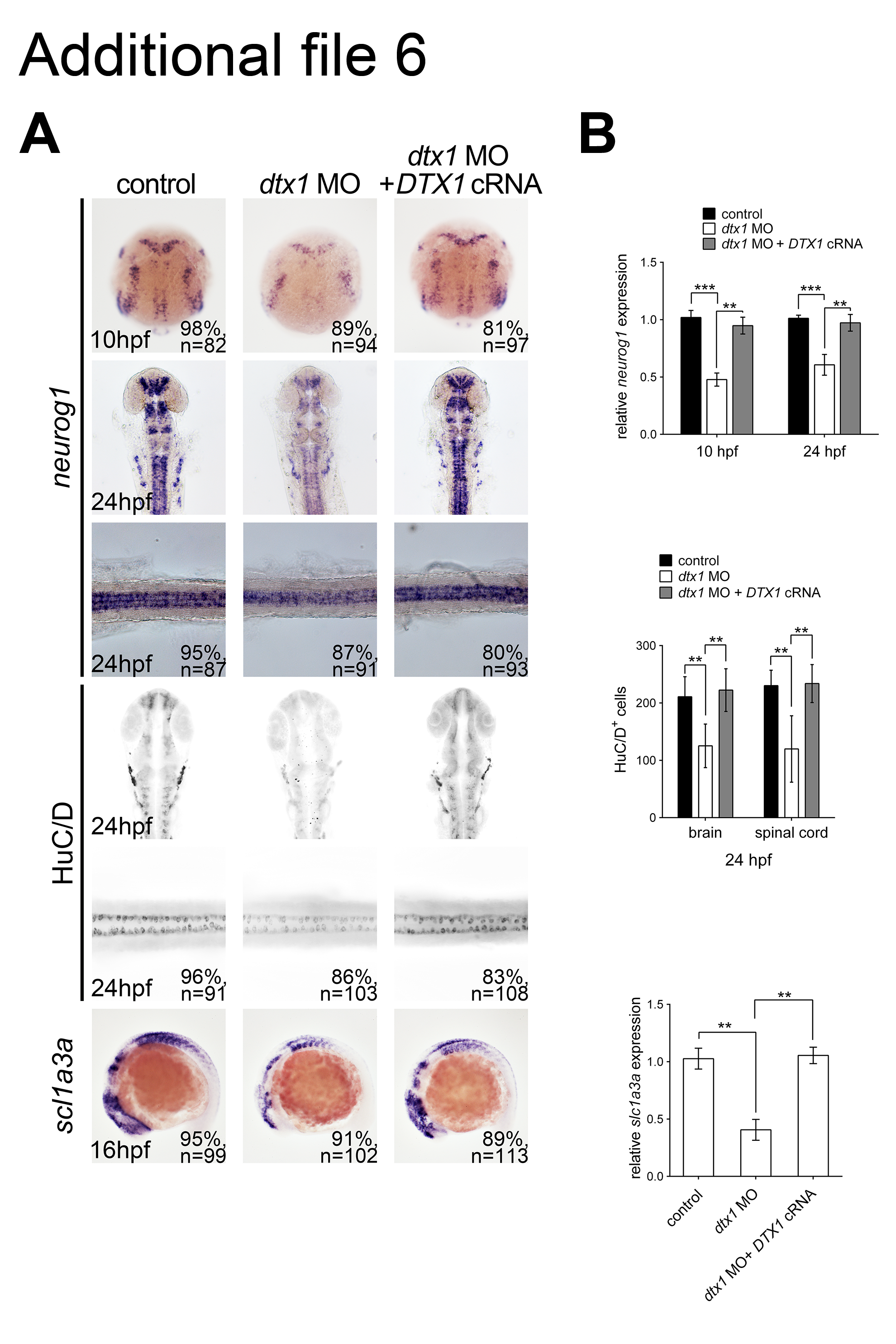

Supplement: Additional file 6: — The phenotypes caused by morpholino injection could be rescued by human DTX1 cRNA. Concomitant injection of human DTX1 cRNA with dtx1 MO1 rescued the neuronal and glial phenotypes caused by Dtx1 knockdown analyzed by in situ hybridization (a) and qPCR (b). (TIF 3052 kb) [file 13064_2015_55_MOESM6_ESM.tif]

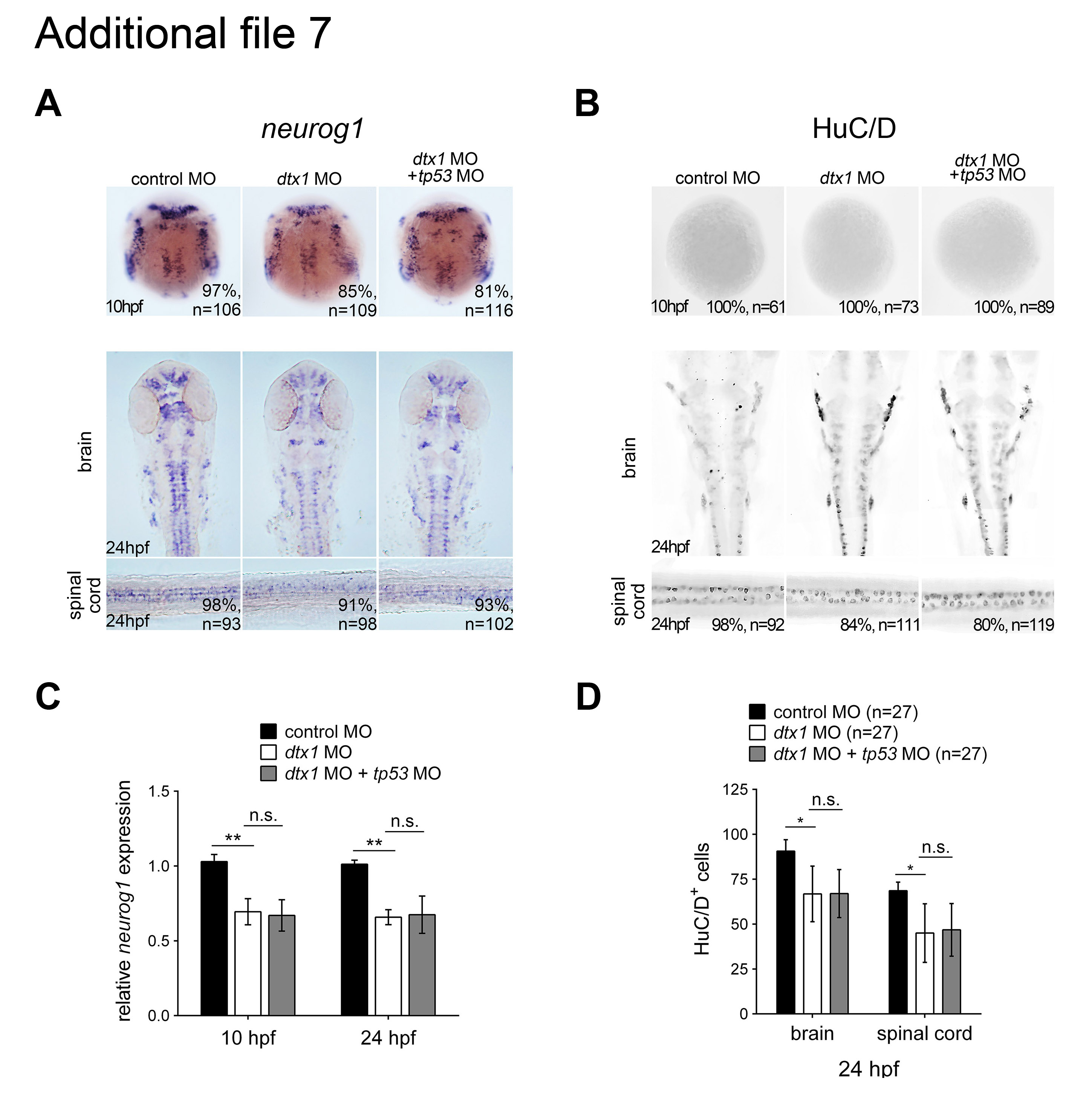

Supplement: Additional file 7: — Phenotypes of Dtx1 morphants were not caused by nonspecific p53 activation. Embryos coinjected with dtx1 MO and tp53 MO were compared with Dtx1 morphants, and we observed no detectable deviation from all markers tested. Compared with the controls, the injection of tp53 MO alone did not show any significant alterations, as shown by in situ hybridization (a), immunohistochemistry (b), qPCR (c), and cell count (D).*, P < 0.05; **, P < 0.01; n.s., nonsignificant. (TIF 2990 kb) [file 13064_2015_55_MOESM7_ESM.tif]

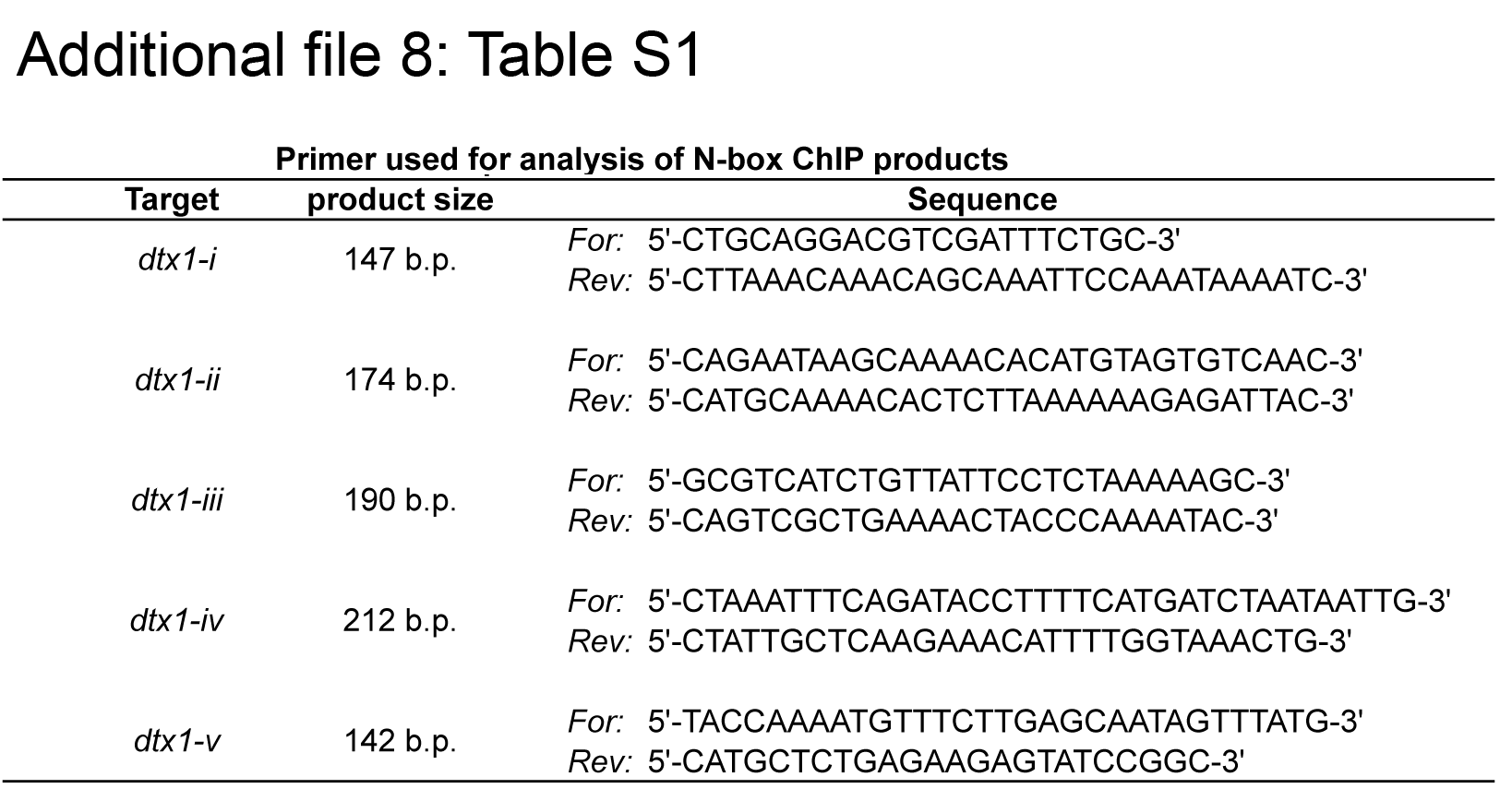

Supplement: Additional file 8: Table S1. — Primers used for ChIP-PCR analysis. (TIF 156 kb) [file 13064_2015_55_MOESM8_ESM.tif]
